# Development of Glycyrrhizic Acid Nanoparticles for Modulating Gastric Ulcer Healing: A Comparative In Vivo Study Targeting Oxidative Stress and Inflammatory Pathways

**DOI:** 10.3390/antiox14080990

**Published:** 2025-08-12

**Authors:** Mody Albalawi, Sahar Khateeb

**Affiliations:** 1 Department of Biochemistry, Faculty of Science, University of Tabuk, Tabuk 71491, Saudi Arabia; ms_albalawi@ut.edu.sa; 2 Biochemistry Division, Department of Chemistry, Faculty of Science, Fayoum University, Fayoum 63514, Egypt

**Keywords:** glycyrrhizic acid, gastric ulcer, nanoparticles, ethanol, oxidative stress, inflammation, *JAK2*/*STAT3*, TGF-β1/Smad3, SIRT1/FOXO1/ PGC-1α

## Abstract

Gastric ulcer (GU) is a common gastrointestinal disorder that impacts quality of life. Currently, several drugs are available for GU treatment, including proton pump inhibitors like omeprazole (OMP); however, their use is limited by numerous potential adverse effects. Glycyrrhizic acid (GLY), a natural anti-inflammatory agent, exhibits promising gastroprotective properties; however, its use is likewise limited by numerous potential adverse effects. This study aimed to synthesize GLY nanoparticles (GLY-NPs) to enhance their therapeutic potential and to comparatively evaluate their efficacy against OMP in an ethanol-induced GU in male Wistar rats. GLY-NPs were synthesized via a hydrothermal method and characterized using TEM, XRD, FTIR, and zeta potential analyses. In vivo, GLY-NPs significantly attenuated gastric mucosal damage compared to OMP, as evidenced by macroscopic and histopathological analyses. Biochemical assays revealed that GLY-NPs markedly improved antioxidant defenses by elevating SOD, catalase, and glutathione peroxidase activities while reducing MDA levels, surpassing the effects of OMP. Furthermore, GLY-NPs modulated inflammatory responses by downregulating p38 MAPK, NF-κB, and TNF-α expression, concomitant with upregulation of the anti-inflammatory cytokine IL-10. Mechanistic insights indicated that GLY-NPs favorably regulated key signaling pathways implicated in gastric mucosal protection, including suppression of the *JAK2*/*STAT3* and TGF-β1/Smad3 pathways, alongside activation of the SIRT1/FOXO1/PGC-1α axis. In conclusion, these findings indicate that GLY-NPs offer higher gastroprotective effects relative to traditional OMP therapy through comprehensive modulation of oxidative stress, inflammation, and molecular signaling pathways. This study highlights GLY-NPs as a potent nanotherapeutic candidate for the effective management of GU.

## 1. Introduction

Gastric ulcers (GUs) are a significant public health concern that negatively affects health and quality of life, with a lifetime frequency of 5–10% in the general population [1,2]. GUs are a prevalent gastrointestinal disorder characterized by the erosion of the stomach lining, leading to considerable pain and discomfort [3]. GUs might develop from excessive alcohol consumption, pathogenic infections, non-steroidal anti-inflammatory medicines, and stress [4]. Ethanol is the predominant alcohol utilized for generating GUs in rats. The ethanol-induced GU model generates oxidative stress by enhancing reactive oxygen species (ROS) production, increasing malondialdehyde (MDA) levels, elevating inflammatory cytokines such as tumor necrosis factor-alpha (TNF-α), and reducing the activity of antioxidant enzymes, including catalase and superoxide dismutase (SOD) [5].

Oxidative stress triggers signaling pathways in stomach cells that affect inflammation and tissue repair. Among these pathways, mitogen-activated protein kinases (MAPKs) constitute significant intracellular signal transduction pathways [6]. The activated MAPK cascade stimulates the nuclear factor kappa B (NF-Κb) cascade [7]. Further, NF-κB responds promptly to stress and inflammatory stimuli and is essential for the pathogenesis of GU [8]. Furthermore, the activation of pro-inflammatory cytokines results in the suppression of anti-inflammatory cytokines, such as interleukin-10 (IL-10) and transforming growth factor-beta (TGF-β), which are vital for tissue remodeling and repair and play a crucial role in regulating the immune response [9]. While TGF-β plays a protective role in safeguarding inflammation, it has also been identified as a key mediator of fibrosis [10]. It significantly contributes to the advancement of fibrosis in the gut and other organs [11], primarily through the activation of the Smad signaling cascade. TGF-β1 can additionally activate the Janus kinase 2 (JAK2)/signal transducer and activator of transcription 3 (STAT3) pathway through a non-classical mechanism [12]. JAK is a non-receptor tyrosine protein kinase activated by several cytokines that triggers downstream target genes through STAT, thus modulating numerous cellular processes [13].

In contrast, multiple mechanisms augment gastroprotection against oxidative damage by mitigating the inflammatory response. Sirtuin 1 (SIRT1) participates in various physiological and pathological signaling pathways [14]. SIRT1 alleviates cellular oxidative stress [15]. Moreover, SIRT1 can interact with several signaling molecules, including forkhead box protein O1 (FOXO1), peroxisome proliferator-activated receptor gamma coactivator alpha (PGC-1α), and NF-κB, therefore modulating oxidative stress and inflammation [16,17]. SIRT1 enhances PGC-1α, hence increasing the mitochondrial synthesis of antioxidant genes, including glutathione peroxidase (GSH-Px), catalase, and SOD [18].

A wide range of drugs are available for gastrointestinal therapy, including proton pump inhibitors [19]. However, the practical application of these drugs is limited due to their inadequate efficacy against GUs and numerous potential side effects [20]. Omeprazole (OMP) is a frequently employed proton pump inhibitor for the management of GUs by diminishing acid output; nonetheless, it is associated with several adverse effects [21]. Thus, there is an imperative necessity of exploring alternative, natural sources of gastroprotective drugs that are both effective and safe. Conventional medicines, including medicinal plants and herbal remedies, have become extensively utilized as alternative treatments for many diseases, particularly gastrointestinal disorders [22,23]. They are known for their high antioxidant, anti-inflammatory, and anti-apoptotic properties and possess the capacity to offer significant gastrointestinal protection [5]. Exploiting these natural resources can develop novel therapies to enhance health [24].

Glycyrrhizic acid (GLY) constitutes the major component of licorice [25]. It is recognized for its anti-inflammatory, antiviral, antioxidant, and anticancer properties [26,27]. Previous studies have shown the potential efficacy of GLY as a therapy for peptic ulcers [28]. Nonetheless, its application is constrained by inadequate bioavailability and poor solubility [29,30]. Consequently, employing suitable ways to augment the solubility of GLY will significantly raise its bioavailability and improve its therapeutic efficacy in treating inflammatory disorders. Currently, nanomedicine encompasses a broader range of applications, including disease evaluation and treatment [31,32,33]. It has the potential to address bioavailability challenges by enhancing the efficacy of plant-derived products, decreasing required dosage, mitigating unwanted effects, and augmenting biological activity. Diverse nanocarrier technologies have demonstrated encouraging outcomes in enhancing GLY delivery and bioavailability [34]. However, to our knowledge, its efficacy in treating ethanol-induced GUs has not been investigated, nor has its impact on critical signaling pathways, including JAK2/STAT3, TGF-β1/Smad3, and SIRT1/FOXO1/PGC-1α, which remains mainly unexplored. The current study aimed to develop GLY nanoparticles (GLY-NPs) to improve their therapeutic potential and to comparatively assess their efficacy against OMP in ethanol-induced GUs in Wistar rats.

## 2. Materials and Methods

### 2.1. Materials

OMP powder was obtained from Sigma (St. Louis, MO, USA). GLY ammonium salt was obtained from Sigma-Aldrich (Catalog# 50531-10G, Lot# BCBC5576V). Diaminobenzidine was obtained from Sigma (DAB; Sigma, St. Louis, MO, USA). The other chemicals employed in the study were of analytical grade.

### 2.2. Preparation of GLY-NPs

GLY-NPs were synthesized using a hydrothermal process as described by Zhao et al. [26]. GLY (10 mg/mL) was solubilized in deionized water (pH = 9.0) and thereafter incubated for 6 h at 185 °C. The samples were centrifuged at 10,000 rpm for 10 min. The supernatant was collected, and the minor precipitate was then eliminated using a dialysis bag (MW 14 kD) to dialyze deionized water for 12 h. Throughout the dialysis procedure, deionized water was substituted every 2 h for obtaining GLY-NPs.

#### 2.2.1. Characterization of GLY-NPs

Particle size and zeta potential were assessed by a Zetasizer Nano ZS (Nano Series HT, Malvern Instruments, Malvern, UK). The morphology of the GLY-NPs was investigated utilizing a transmission electron microscope (TEM, JEOL JEM-1230, JEOL Ltd., Tokyo, Japan). The images were obtained at an accelerating voltage of 120 kV.

The X-ray diffraction (XRD) pattern of the GLY-NPs was examined utilizing a PANalytical X’Pert PRO X-ray diffractometer (PANalytical B.V., Almelo, The Netherlands). The analysis was carried out at a scanning range of 5.0° to 89.99° (2θ) at 30 mA, 40 kV. The presence of functional groups was confirmed utilizing Fourier transform infrared (FTIR) spectroscopy (Nexus 670, Nicolet, Madison, WI, USA). The sample was scanned between 400–4000 cm^−1^.

#### 2.2.2. In Vitro Drug Release

The drug release of the GLY-NPs was assessed using a diffusion method via a dialysis bag (12,000 MW). Briefly, 5 mg of GLY-NPs was encapsulated in a dialysis bag and immersed in 20 mL of phosphate-buffered saline (PBS) at pH 7.4 and 37 °C. At designated time intervals (1–8 h), 2 mL samples were withdrawn and substituted with fresh PBS. The quantity of released drugs was measured using E-2100 UV–Vis spectrophotometry (Peak Instruments Inc., Houston, TX, USA) at 289 nm (Supplementary Figure S1), and the rate of drug release was plotted versus time.

#### 2.2.3. Entrapment Efficiency (EE%)

The EE was assessed by evaluating the free GLY amount in the supernatant produced after centrifuging the GLY-NPs suspension at 12,000 rpm for 30 min. The concentration was assessed using UV–Vis spectrophotometry at 289 nm [35]. The EE (%) was determined utilizing the formula:

$$EE\;(\%)=\frac{Total\;drug-Free\;drug}{Total\;drug}\times 100$$

### 2.3. Induction of GUs and Experimental Grouping

**Figure 1:**
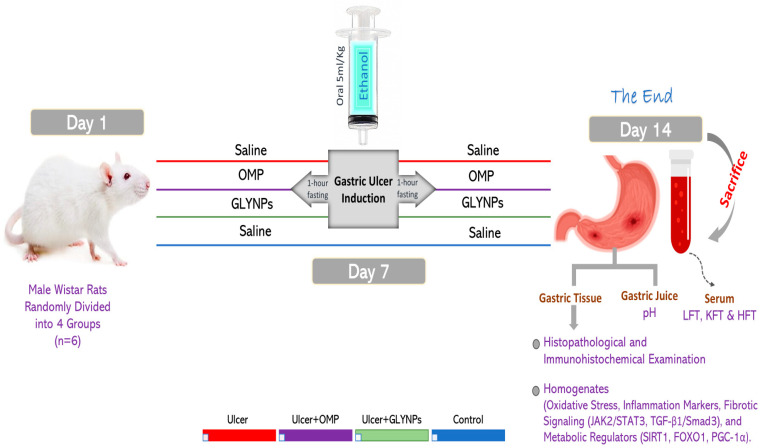
Induction of GUs and experimental grouping. Omeprazole (OMP), glycyrrhizic acid nanoparticles (GLY-NPs), liver function test (LFT), kidney function test (KFT), and hormone function test (HFT).

Twenty-four healthy male Wistar rats, with weights ranging from 200 to 250 gm, were employed. GUs were induced with a single oral dose of absolute ethanol (5 mL/kg) through intragastric gavage [36]. The experimental subjects were randomly allocated to four groups (n = 6). The control group consisted of untreated, normal rats. The ulcer group (UL): rats were administered a single dose of absolute ethanol on day 7 and remained untreated until the end of the experiment on day 14. The Ulcer + OMP group: rats received oral OMP (20 mg/kg) for 7 days [37,38]. On day 7, one hour after OMP therapy, the rats were administered a single dose of absolute ethanol, followed by daily OMP delivery until day 14. The ulcer + GLY-NPs group: rats were administered oral GLY-NPs (10 mg/kg) [39] for 7 days, followed by a dose of absolute ethanol on day 7, one hour post-GLY-NP administration, with GLY-NP treatment persisting until day 14 (Figure 1).

At the end of the 14-day study, rats were weighed and subsequently euthanized after being administered anesthesia. Blood samples have been gathered and centrifuged at 3000 rpm for 5 min at 4 °C to obtain the serum, which was then stored at −20 °C until analysis. The stomachs of the rats were excised, cut along their outer curvature, and gastric fluid subsequently was collected. The fluids were centrifuged to separate the supernatant gastric juice, which was then evaluated for its pH level (pH meter, HANNA, HI 110, Smithfield, RI, USA). Stomach tissues were divided into two sections: one was preserved in 10% formalin for histological and immunohistochemical examination, while the other was utilized to create a gastric homogenate for biochemical evaluation.

#### 2.3.1. Assessment of Gastric Mucosal Damage, Ulcer Index (UI%), and Ulcer Inhibition (%)

Stomachs were digitally imaged for macroscopic evaluation of mucosal damage to identify any hemorrhagic lesions on the glandular mucosa [40]. The images were examined with ImageJ2 software (version 2.16.0; NIH, Bethesda, MD, USA) to quantify the ulceration areas. UI (%) and Ulcer Inhibition (%) were estimated following the methods outlined by Szabo and Hollander [41] and Wu et al. [42], respectively, employing the following formula:

$$UI\;(\%)=\frac{Ulcerated\;area}{Total\;stomach\;area}\times 100$$

$$Ulcer\;Inhibition\;(\%)=\frac{UI\;in\;ulcer\;group-UI\;in\;test\;group}{UI\;in\;ulcer\;group}\times 100$$

#### 2.3.2. Biochemical Analysis

##### Spectrophotometric and Enzyme-Linked Immunosorbent Assay (ELISA)

The activities of alanine aminotransferase (ALT), aspartate aminotransferase (AST), and alkaline phosphatase (ALP) were evaluated using Spectrum Diagnostics (Obour City, Egypt), adhering to the manufacturer’s guidelines. Urea (Cat. No. ab83362, Cambridge, UK) and uric acid (Cat. No. MA-UA, Norcross, GA, USA) levels were measured in serum following the manufacturer’s instructions. The activities of creatinine, catalase, and GSH-Px were evaluated using Bio-diagnostic (Giza, Egypt) in accordance with the manufacturer’s procedure.

Various biomarkers were quantified using commercially available ELISA kits according to the manufacturers’ protocols. IL-10 (Cat. No. SEA056Ra) and TNF-α (Cat. No. SEA133Ra) were measured using kits from Cloud-Clone Corp (Katy, TX, USA). SOD1 (Cat. No. MBS036924), MDA (Cat. No. MBS268427), somatostatin (Cat. No. MBS260025), gastrin (Cat. No. MBS494300), P38MAPK (Cat. No. MBS720509), FOXO1 (Cat. No. MBS749342), PGC1α (Cat. No. MBS775129), TGF-β1 (Cat. No. MBS824788), Smad3/MADH3 (Cat. No. MBS2516123), SIRT1 (Cat. No. MBS2600246), and NFκB (Cat. No. MBS453975) were analyzed using MyBioSource kits (San Diego, CA, USA), following the manufacturer’s protocol.

##### RNA Extraction and Quantitative RT-PCR Analysis

Total RNA was isolated from tissue lysate utilizing the Direct-zol RNA Miniprep Plus (Cat# R2072, Zymo Research Corp., Tustin, CA, USA), and its quantity and quality were subsequently assessed using a Beckman dual spectrophotometer (Brea, CA, USA). Thermal cycling was performed with a StepOne Real-Time PCR System (Applied Biosystems, Waltham, MA, USA). Relative gene expression was assessed via the 2^−ΔΔCt^ method, normalized to *GAPDH*. The qRT-PCR primers for *STAT3*, *JAK2*, and *GAPDH* are listed in Table 1.

#### 2.3.3. Histopathological and Immunohistochemical (IHC) Assay

Gastric tissues were dehydrated in ascending concentrations of alcohol, cleared in xylene, embedded in paraffin wax, and sectioned to a thickness of 5 μm. Prepared slide sections were stained with hematoxylin and eosin (H&E) and analyzed using a digital light microscope (Olympus XC30, Tokyo, Japan). Histopathological alterations in the gastric tissue were evaluated semi-quantitatively in five randomly chosen high-power fields (20× magnification) per section. The assessed criteria encompassed epithelium loss, necrosis, edema, and infiltration of inflammatory cells. Each parameter was evaluated for severity using a 4-point scale: 0 = normal, 1 = mild, 2 = moderate, and 3 = severe deviations. This grading system was derived from previously described histological scoring methods for stomach damage [43], with minor modifications.

For immunohistochemical examination, the deparaffinized and rehydrated sections were subjected to incubation in 3% H_2_O_2_, followed by incubation with rabbit monoclonal anti-PTEN (sc-7974), anti-PI3K (sc-365404), and anti-AKT (sc-5298) as primary antibodies. Diaminobenzidine was incorporated to demonstrate the immunological response. The immunohistochemistry staining for PTEN, PI3K, and AKT was semi-quantitatively assessed in 10 high-power microscopic areas (40×), as outlined by Hegazy et al. [44]. The assessment of gastric mucosa was predicated on two primary criteria: color intensity and the percentage of positively stained cells. A grading scale from 0 to 3 was employed for color intensity, where 0 indicated no staining, 1 denoted weak staining, 2 signified moderate staining, and 3 represented intense staining. For the assessment of the percentage of positively stained cells in high-power fields, a grading system ranging from 0 to 3 was employed, where grade 0 signified 0%, grade 1 denoted <30%, grade 2 represented 30–70%, and grade 3 showed >70%. The combination of these two criteria creates the total immunoreactivity score.

### 2.4. Statistical Analysis

The statistical analysis for this study was performed utilizing GraphPad Prism Software version 9.5.1 (GraphPad Software Inc., San Diego, CA, USA). Experimental data were reported as mean ± SEM (n = 6). Group differences were evaluated using a one-way analysis of variance (ANOVA) accompanied by a post hoc Tukey test, with a significance level of *p* < 0.05. The Shapiro–Wilk test for normality was employed to assess the data distribution for histopathological and immunohistochemical scoring. Given the non-normal distribution of the score data, a nonparametric statistical analysis employing the Kruskal–Wallis test was performed just for this parameter, followed by Dunn’s multiple comparisons test.

## 3. Results

### 3.1. Zeta Potential, Average Particle Size Evaluation, and TEM Imaging of GLY-NPs

**Figure 2:**
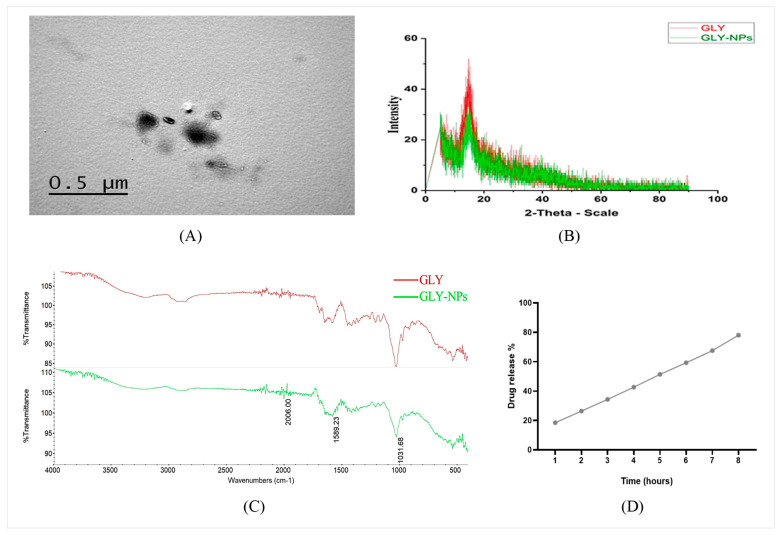
Structural characterization of the GLY-NPs. (**A**) The TEM image of GLY-NPs showed spherical to oval-shaped particles with sizes ranging between 50 and 135 nm (scale bar, 500 nm); (**B**) XRD patterns of GLY and GLY-NPs; (**C**) FTIR spectrum of GLY and GLY-NPs; and (**D**) in vitro release profile of GLY-NPs.

The GLY-NP exhibited a Z-average diameter of 136.3 nm, with a polydispersity index (PDI) of 0.489 and a zeta potential of −19.2 mV, indicating moderate variation in particle size distribution and acceptable formulation stability (Details are provided in Supplementary Figures S2 and S3). TEM was employed to investigate the morphology of GLY-NPs (Figure 2A). The morphology of GLY-NPs can be described as spherical to oval-shaped, with sizes ranging between 50 and 135 nm.

### 3.2. XRD and FTIR Spectra Analysis of GLY and GLY-NPs

The XRD diffractograms of GLY and the GLY-NPs are depicted in Figure 2B. The XRD study demonstrated pronounced sharp peaks at 14.5° (2θ) in the pure GLY spectrum, signifying its crystalline structure. In contrast, the GLY-NPs pattern showed a less intense peak, suggesting a significant reduction in crystallinity. This reduction may be attributed to the nanosizing process and the possible formation of an amorphous or partially amorphous structure during nanoparticle preparation.

The FTIR spectra of GLY and GLY-NPs depicted in Figure 2C demonstrate extensive structural similarity, signifying the retention of the parent compound’s functional groups post-nano formulation. Both spectra display significant peaks indicative of C–H stretching (~2862–2900 cm^−1^), C=O/C=C stretching (~1589 cm^−1^), and pronounced C–O stretching (~1032–1033 cm^−1^), hence affirming the preservation of triterpenoid and glycosidic structures. Notably, the appearance of an overtone band at 2006 cm^−1^ in GLY-NPs suggests subtle changes in molecular packing due to particle size reduction.

### 3.3. In Vitro Release Profile and EE% of GLY-NPs

The in vitro release profile of GLY from the created GLY-NPs demonstrated sustained and controlled release behavior over an 8 h period, as illustrated in Figure 2D. The percentage of drug release progressively ascended over time, from 18.5 ± 0.5% at 1 h to 78.0 ± 1.0% at 8 h. This release pattern is beneficial for maintaining therapeutic medication levels over an extended period and is particularly effective for the treatment of GUs. Moreover, the GLY-NPs exhibited high EE%, with values of 98.10%, 98.33%, and 98.40% for three independent batches. This yielded a mean EE of 98.28 ± 0.23% (mean ± SD, n = 3), demonstrating great batch-to-batch consistency. The minimum standard deviation (0.23%) signifies the precise reproducibility of the nanofabrication process.

### 3.4. Effects of GLY-NPs and OMP on Body Weight, Stomach Weight, Stomach Coefficient, and Gastric pH in Ethanol-Induced GUs in Rats

**Figure 3:**
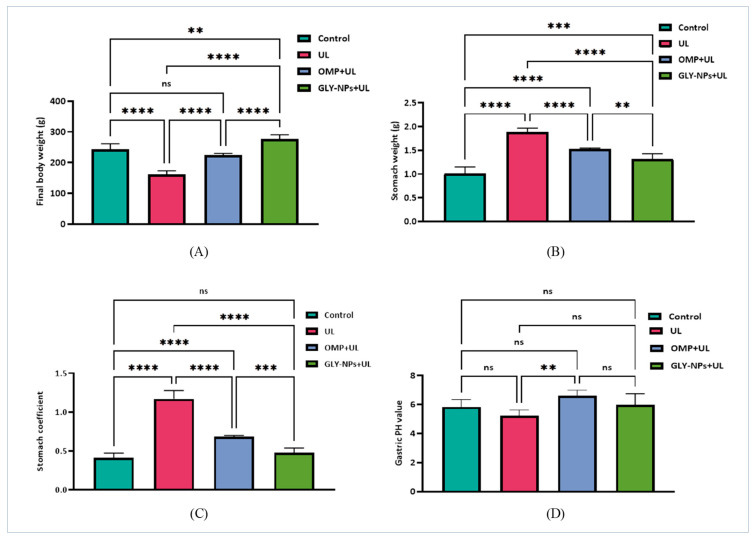
Effects of GLY-NPs and OMP on (**A**) final body weight, (**B**) stomach weight, (**C**) stomach coefficient, and (**D**) gastric pH in ethanol-induced GUs. The data represent the mean ± SEM (n = 6). Statistical comparisons were performed using one-way ANOVA followed by Tukey’s post hoc test. **** *p* < 0.0001, *** *p* < 0.001, ** *p* < 0.01, ns = not significant.

The administration of ethanol in the UL group resulted in a substantial decrease in the final body weight of rats (162.3 ± 4.52 g) in comparison to the control group (243.5 ± 7.36 g; *p* < 0.0001), indicating systemic toxicity and stress caused by gastric damage. Administration with OMP resulted in a partial restoration of body weight to 224.3 ± 2.36 g; however, the increase was not statistically significant when compared to controls (*p* = 0.0823). Notably, GLY-NPs administration not only averted weight loss but also significantly elevated the end body weight to 276.7 ± 5.73 g in comparison to both the UL and control groups (*p* = 0.0014 and *p* < 0.0001, respectively), signifying improved general health and recovery (Figure 3A).

Regarding stomach weight, the UL group demonstrated a substantial increase in stomach weight (1.888 ± 0.033) compared to the control group (1.012 ± 0.057; *p* < 0.0001), likely attributable to edema and inflammatory infiltration. OMP administration significantly decreased stomach weight to 1.527 ± 0.009 (*p* < 0.0001 vs. the UL group), while GLY-NPs further reduced stomach weight to 1.315 ± 0.047, which was significantly lower than both the UL group and OMP-treated rats (*p* = 0.0002 and *p* = 0.0073, respectively), as displayed in Figure 3B. Similarly, the stomach coefficient significantly increased in the UL group (1.169 ± 0.044) relative to controls (0.416 ± 0.023; *p* < 0.0001), signifying pathological gastric enlargement. The administration of OMP significantly decreased the stomach coefficient to 0.681 ± 0.007 (*p* < 0.0001 compared to UL), whereas GLY-NPs further reduced it to 0.478 ± 0.025, which is comparable to control levels (*p* = 0.4303) and significantly lower than both the ulcer and OMP groups (*p* < 0.0001 and *p* = 0.0003, respectively), thereby underscoring the superior gastroprotective efficacy of GLY-NPs (Figure 3C).

The administration of ethanol in the UL group resulted in a moderate decrease in gastric juice pH relative to the control group; nonetheless, this decrease did not achieve statistical significance (*p* = 0.3005). Treatment with OMP markedly increased gastric pH relative to the UL group (*p* = 0.0040), signifying effective acid suppression. While GLY-NPs appeared to elevate the pH compared to the UL group, the difference was not statistically significant (*p* = 0.1526). Likewise, comparisons between GLY-NPs + UL and OMP + UL (*p* = 0.2778), as well as between GLY-NPs + UL and the control group (*p* = 0.9728), did not demonstrate significant differences (Figure 3D). These findings indicate that although GLY-NPs produce a slight alkalinizing impact, it is less effective than that of OMP.

### 3.5. Macroscopic Evaluation of Gastric Mucosa, UI %, and Ulcer Inhibition (%) in Ethanol-Induced GUs in Rats

**Figure 4:**
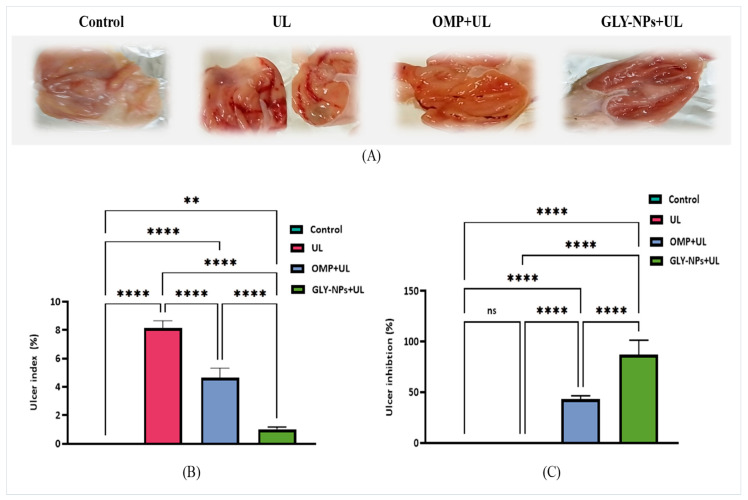
(**A**) The appearance and morphology of stomach tissue from each group, (**B**) ulcer index, and (**C**) ulcer inhibition (%). The data represent the mean ± SEM (n = 6). Statistical comparisons were performed using one-way ANOVA followed by Tukey’s post hoc test. **** *p* < 0.0001, ** *p* < 0.01, ns = not significant.

The macroscopic assessment of the gastric mucosa demonstrated distinct variations across the experimental groups. The control group exhibited normal, smooth gastric mucosa devoid of obvious lesions. Conversely, the UL group displayed significant hemorrhagic lesions, edema, and prominent ulceration, indicative of serious mucosal damage caused by ethanol. Administration with OMP revealed significant enhancement, characterized by decreased hemorrhage and ulcer sizes, signifying partial gastroprotection. GLY-NPs administration exhibited significantly preserved stomach mucosa, characterized by minor ulcerative lesions and less edema, as shown in Figure 4A.

Furthermore, as illustrated in Figure 4B, the UL group exhibited a considerable increase in UI% compared to the controls (8.162 ± 0.2055% vs. 0.000 ± 0.000%; *p* < 0.0001), indicating substantial gastric mucosal damage. Administration of OMP significantly decreased UI% to 4.648 ± 0.2735% (*p* < 0.0001 vs. UL). Also, GLY-NPs administration caused a further drop in UI% to 1.020 ± 0.0639%, which was markedly lower than the UL and OMP + UL groups (*p* < 0.0001). Regarding ulcer inhibition (%), OMP administration significantly elevated it to 43.10 ± 1.414% (*p* < 0.0001 vs. UL). In addition, the GLY-NPs administration exhibited elevated ulcer inhibition (%) of 87.50 ± 5.667% (*p* < 0.0001 compared to UL and OMP + UL), indicating therapeutic efficacy (Figure 4C). These findings confirm that GLY-NPs provide enhanced gastroprotection and accelerate gastric mucosal repair more effectively than OMP.

### 3.6. Impact of Ethanol-Induced GUs and Treatments on Renal Biomarkers and Hepatic Enzyme Levels

**Figure 5:**
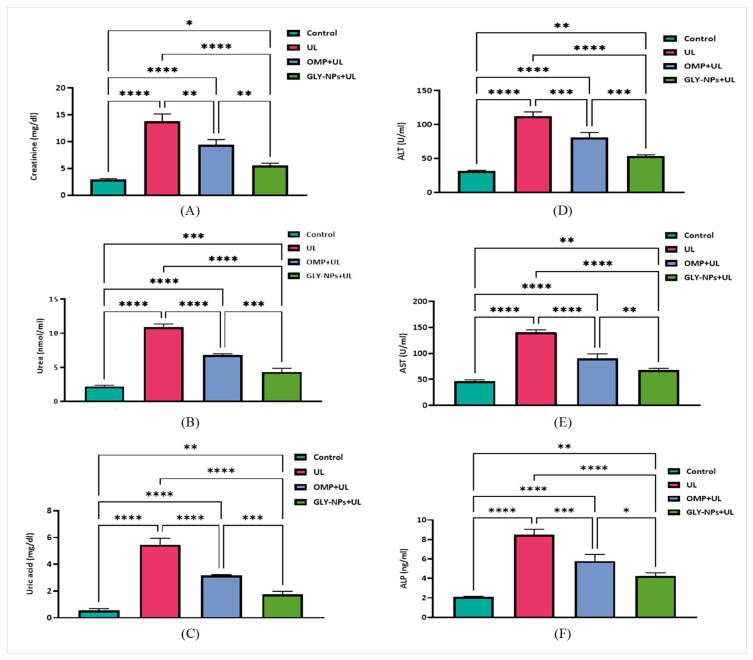
Effect of GLY-NPs and OMP on serum renal function biomarkers and hepatic enzyme activities in the ethanol-induced GUs model: (**A**) creatinine (mg/dL), (**B**) urea (nmol/mL), (**C**) uric acid (mg/dL), (**D**) ALT (U/L), (**E**) AST (U/L), and (**F**) ALP (U/L). Data are presented as mean ± SEM (n = 6). Statistical comparisons were performed using one-way ANOVA followed by Tukey’s post hoc test. * *p* < 0.05, ** *p* < 0.01, *** *p* < 0.001, **** *p* < 0.0001.

Ethanol-induced GUs generated a marked deterioration in renal function. Creatinine levels increased markedly from 2.90 ± 0.12 mg/dL in the control group to 13.75 ± 0.80 mg/dL in ulcerated rats (*p* < 0.0001) but were significantly reduced to 9.43 ± 0.55 mg/dL and 5.55 ± 0.25 mg/dL in the OMP + UL and GLY-NPs + UL groups, respectively. The GLY-NPs treatment produced a statistically significant improvement compared to OMP (*p* = 0.0028) (Figure 5A).

Urea concentrations rose from 0.565 ± 0.072 nmol/mL in controls to 5.46 ± 0.28 nmol/mL in the UL group (*p* < 0.0001). Both treatments significantly ameliorated urea elevation, with levels reduced to 3.18 ± 0.02 and 1.76 ± 0.13 nmol/mL in the OMP + UL and GLY-NPs + UL groups, respectively (*p* < 0.0001 for both vs. UL). Importantly, GLY-NPs showed better efficacy than OMP (*p* = 0.0003) (Figure 5B).

A similar trend was observed with uric acid, which rose dramatically in ulcer-induced rats (10.87 ± 0.28 mg/dL) compared to controls (2.19 ± 0.10 mg/dL, *p* < 0.0001). This elevation was significantly attenuated by both treatments, with uric acid levels reduced to 6.79 ± 0.12 and 4.30 ± 0.32 mg/dL in the OMP + UL and GLY-NPs + UL groups, respectively. Once again, GLY-NPs demonstrated a significantly stronger effect than the OMP (*p* = 0.0010) (Figure 5C).

Regarding liver function, ALT activity significantly increased from 31.47 ± 0.64 U/mL in the control group to 111.9 ± 3.91 U/mL in the UL group (*p* < 0.0001). Administration with OMP decreased ALT to 80.77 ± 4.38 U/mL (*p* = 0.0004 compared to UL), while GLY-NPs administration dramatically reduced ALT levels to 53.34 ± 1.18 U/mL, which was statistically lower than OMP (*p* = 0.0009) and considerably different from the control (*p* = 0.0039) (Figure 5D).

AST levels increased from 46.77 ± 1.45 U/mL in the control group to 140.8 ± 2.67 U/mL in the ulcerated rats (*p* < 0.0001). The OMP + UL and GLY-NPs + UL groups exhibited significant decreases to 90.15 ± 5.27 and 67.84 ± 1.99 U/mL, respectively (*p* < 0.0001 compared to UL; *p* = 0.0051 for GLY-NPs + UL compared to OMP + UL) (Figure 5E). A notable increase in ALP levels from 2.10 ± 0.03 ng/mL in the control group to 8.52 ± 0.31 ng/mL in the UL group was detected (*p* < 0.0001). Both treatments substantially diminished this increase, with ALP levels decreased to 5.79 ± 0.39 in OMP + UL and 4.27 ± 0.18 ng/mL in GLY-NPs + UL. The latter demonstrated a statistically significant efficiency compared to OMP (*p* = 0.0156) (Figure 5F).

### 3.7. Impact of GLY-NPs and OMP on Gastrin and Somatotropin Hormone Levels in Ethanol-Induced GUs in Rats

Ethanol-induced gastric ulceration was associated with a significant elevation in serum gastrin and somatotropin levels relative to control rats (*p* < 0.0001 for both hormones). The administration of OMP significantly reduced gastrin and somatotropin levels relative to the UL group (*p* < 0.0001). Meanwhile, the GLY-NPs administration exhibited a much greater decrease in gastrin and somatotropin levels compared to the UL group and OMP + UL groups (*p* < 0.0001), as displayed in Table 2.

### 3.8. Protective and Therapeutic Effects of Treatments on Oxidative Stress Biomarkers and Antioxidant Levels

**Figure 6:**
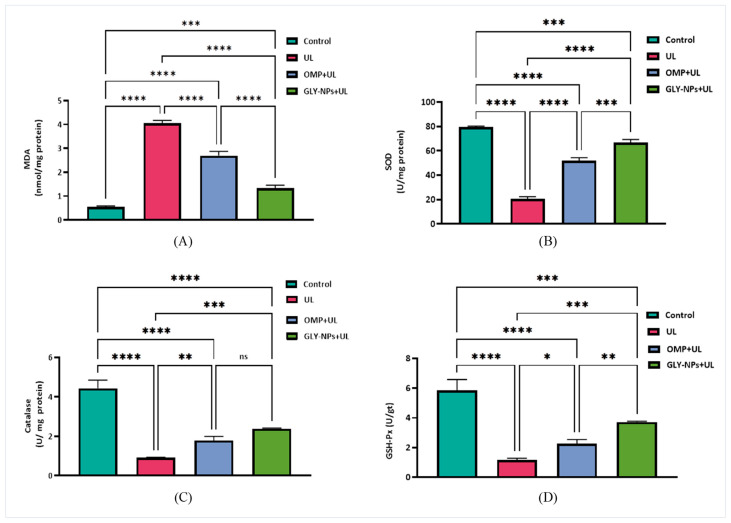
Effect of GLY-NPs and OMP on oxidative stress biomarkers and antioxidant levels in tissue homogenates in ethanol-induced GUs in rats: (**A**) MDA (nmol/mg protein), (**B**) SOD (U/mg protein), (**C**) Catalase (U/mg protein), and (**D**) GSH-Px (U/g tissue). Data are presented as mean ± SEM (n = 6). One-way ANOVA followed by Tukey’s post hoc test. * *p* < 0.05, ** *p* < 0.01, *** *p* < 0.001, **** *p* < 0.0001, and ns = not significant.

Ethanol-induced GUs resulted in a significant increase in MDA levels and a simultaneous reduction in endogenous antioxidant enzymes, such as SOD, catalase, and GSH-Px, in the UL group relative to the control (*p* < 0.0001), while the administration of OMP and GLY-NPs elevated SOD, catalase, and GSH-Px levels (Figure 6). Furthermore, our findings revealed that GLY-NPs were more effective than OMP in this context. These findings collectively indicate that GLY-NPs exert a protective role by mitigating oxidative stress and enhancing enzymatic antioxidant defense, albeit with varying efficacy compared to the reference drug OMP.

### 3.9. Modulatory Effects of GLY-NPs and OMP on p38MAPK, NF-κB, and Inflammatory Cytokines in Ethanol-Induced GUs in Rats

**Figure 7:**
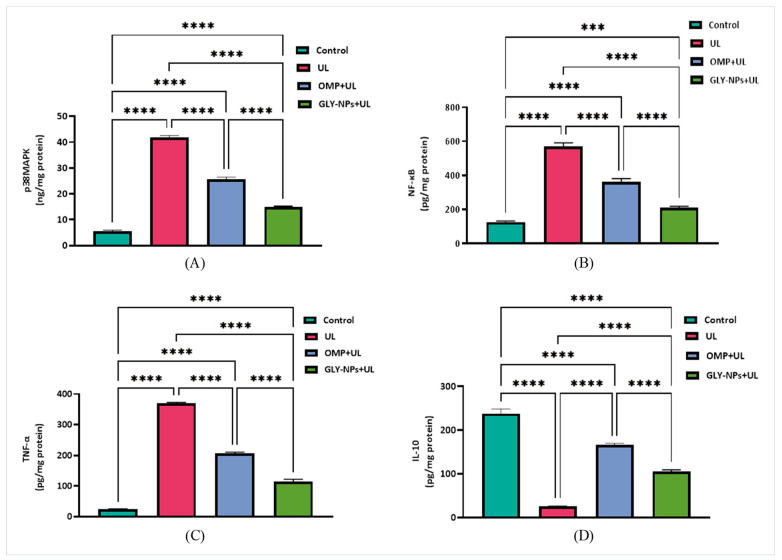
Effects of GLY-NPs and OMP on (**A**) p38MAPK, (**B**) NF-κB, (**C**) TNF-α, and (**D**) IL-10 in tissue homogenates in ethanol-induced GUs in rats. Data expressed as mean ± SEM (n = 6). Statistical comparisons were performed using one-way ANOVA followed by Tukey’s post hoc test. *** *p* < 0.001, **** *p* < 0.0001.

The p38MAPK levels were markedly increased in the UL group at 41.90 ± 0.40 ng/mg protein, signifying a substantial increase relative to the control group (5.51 ± 0.27 ng/mg protein; *p* < 0.0001), demonstrating the activation of inflammatory signaling pathways subsequent to ethanol-induced gastric injury. Treatment with OMP markedly decreased p38MAPK levels to 25.63 ± 0.50 ng/mg protein (*p* < 0.0001 vs. UL), whereas GLY-NPs induced a more substantial reduction to 14.96 ± 0.16 ng/mg protein, significantly lower than both the UL and OMP-treated groups (*p* < 0.0001) (Figure 7A). Likewise, NF-κB levels were significantly elevated in the UL group (571.2 ± 11.32 pg/mg protein) compared to controls (125.0 ± 3.85 pg/mg protein; *p* < 0.0001). OMP treatment diminished NF-κB expression to 362.2 ± 10.33 pg/mg protein (*p* < 0.0001 vs. UL), while GLY-NPs further decreased it to 209.3 ± 5.11 pg/mg protein, significantly lower than both UL and OMP + UL groups (*p* < 0.0001 and *p* = 0.0004, respectively) (Figure 7B), underscoring the potent anti-inflammatory efficacy of GLY-NPs.

TNF-α was significantly increased in the UL group (369.5 ± 2.00 pg/mg protein) compared to the control group (23.17 ± 1.17 pg/mg protein; *p* < 0.0001). Both OMP and GLY-NPs administration dramatically decreased TNF-α levels to 205.9 ± 2.55 and 114.5 ± 4.17 pg/mg protein, respectively, with GLY-NPs exhibiting a pronounced anti-inflammatory impact (*p* < 0.0001 vs. OMP + UL) (Figure 7C). In contrast, IL-10 levels significantly decreased in the UL to 25.56 ± 0.74 pg/mg protein, in contrast to the control group (237.8 ± 6.02 pg/mg protein; *p* < 0.0001). Treatment with OMP partially restored IL-10 levels to 166.3 ± 2.07 pg/mg protein, but GLY-NPs dramatically elevated IL-10 concentration to 104.8 ± 2.48 pg/mg protein (Figure 7D).

### 3.10. Modulation of TGF-β1/Smad3 and JAK2/STAT3 Signaling Pathway by GLY-NPs and OMP in Ethanol-Induced GUs Model

**Figure 8:**
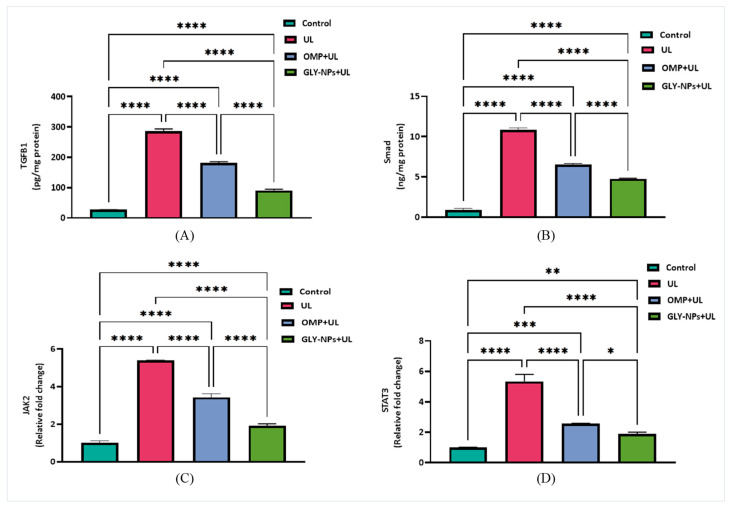
Effects of GLY-NPs and OMP on (**A**) TGF-β1, (**B**) Smad3, (**C**) *JAK2*, and (**D**) *STAT3* gene expression (relative fold change) in tissue homogenates in ethanol-induced GUs in rats. Data are presented as mean ± SEM (n = 6). Statistical analysis was performed using one-way ANOVA followed by Tukey’s post hoc test. * *p* < 0.05, ** *p* < 0.01, *** *p* < 0.001, **** *p* < 0.0001.

TGF-β1 was markedly elevated in the UL group (286.8 ± 3.95 pg/mg protein) compared to the control group (27.14 ± 0.14 pg/mg protein; *p* < 0.0001). OMP treatment reduced TGF-β1 levels to 180.7 ± 2.82 pg/mg protein, whereas GLY-NPs dropped it further, to 90.83 ± 2.41 pg/mg protein, with all intergroup comparisons being highly significant (*p* < 0.0001) (Figure 8A). The results indicate that GLY-NPs efficiently reduce fibrotic reactions caused by ethanol, surpassing the preventive effects of conventional OMP therapy. The Smad3 levels in the UL group significantly increased to 10.85 ± 0.14 ng/mg protein, in contrast to the control group (0.91 ± 0.09 ng/mg protein; *p* < 0.0001). Treatment with OMP dramatically decreased Smad3 expression to 6.56 ± 0.07 ng/mg protein, whereas GLY-NPs further diminished Smad levels to 4.75 ± 0.05 ng/mg protein, both significantly differing from UL (*p* < 0.0001) and demonstrating greater efficacy for GLY-NPs compared to OMP (*p* < 0.0001) (Figure 8B).

Regarding *JAK2/STAT3*, overexpression of both *JAK2* and *STAT3* was detected in the UL group relative to the control (*p* < 0.0001), with expression levels rising nearly 5.4-fold for each marker. Treatment with OMP slightly suppressed this activation, decreasing *JAK2* expression to 3.43 ± 0.11 and *STAT3* to 2.59 ± 0.01 (*p* = 0.0001 vs. UL). Also, GLY-NPs administration resulted in a significant reduction in *JAK2* to 1.92 ± 0.06 and *STAT3* to 1.90 ± 0.06 (*p* = 0.0001 vs. UL). Moreover, GLY-NPs demonstrated a markedly stronger inhibition of *STAT3* expression compared to OMP (*p* = 0.0263) (Figure 8C,D), suggesting a possibly enhanced modulatory effect of GLY-NPs on inflammation-related signaling.

### 3.11. Restorative Effects of GLY-NPs and OMP on SIRT1, FOXO1, and PGC-1α Levels in Ethanol-Induced GUs

**Figure 9:**
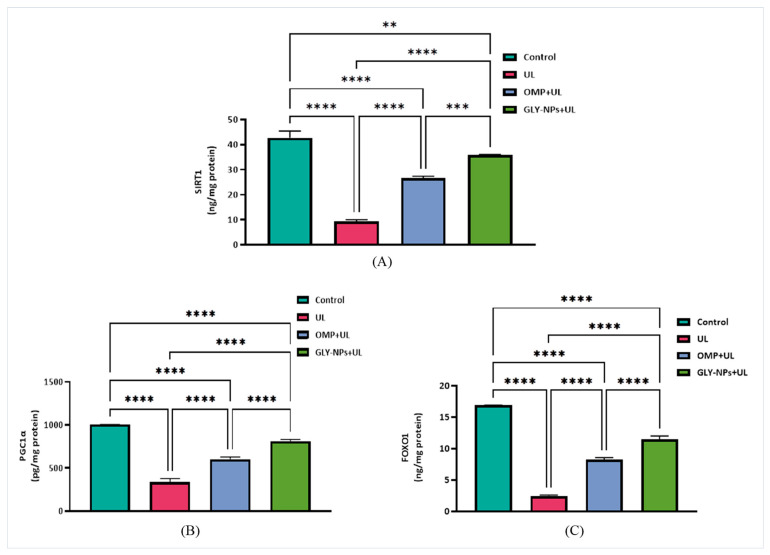
Effects of GLY-NPs and OMP on (**A**) SIRT1, (**B**) PGC-1α, and (**C**) FOXO1 levels in tissue homogenate in ethanol-induced GUs. Data are presented as mean ± SEM (n = 6). Statistical analysis was performed using one-way ANOVA followed by Tukey’s post hoc test. **** *p* < 0.0001, *** *p* < 0.001, ** *p* < 0.01.

For SIRT1, the UL group exhibited a significant decline (9.30 ± 0.40 ng/mg protein) compared to the control (42.70 ± 1.57 ng/mg protein; *p* < 0.0001). OMP partially restored SIRT1 levels to 26.69 ± 0.42 ng/mg protein, whereas GLY-NPs accomplished a nearly complete restoration to 35.78 ± 0.19 ng/mg protein, demonstrating significant differences from both the UL (*p* = 0.0018) and OMP + UL groups (*p* = 0.0003) (Figure 9A). Similarly, PGC-1α exhibited significant downregulation in the UL group (336.5 ± 24.09 pg/mg protein) compared to the control group (1004 ± 0.37 pg/mg protein; *p* < 0.0001). OMP administration elevated PGC-1α levels to 602.7 ± 14.88 pg/mg protein (*p* = 0.0001 vs. UL), whereas GLY-NPs further augmented PGC-1α expression to 812.9 ± 11.10 pg/mg protein (*p* = 0.0001 vs. UL) (Figure 9B).

Moreover, FOXO1 levels in the UL group were significantly diminished to 2.46 ± 0.09 ng/mg protein, in contrast to the control group, which exhibited levels of 16.92 ± 0.01 ng/mg protein (*p* < 0.0001). Treatment with OMP partially restored FOXO1 expression to 8.28 ± 0.17 ng/mg protein, while GLY-NPs induced a more pronounced recovery to 11.52 ± 0.29 ng/mg protein, with both demonstrating statistically significant enhancements relative to UL (*p* < 0.0001). In addition, GLY-NPs markedly surpassed OMP in the restoration of FOXO1 (*p* < 0.0001) (Figure 9C). The data collectively demonstrate that GLY-NPs substantially mitigate the ethanol-induced downregulation of FOXO1, PGC-1α, and SIRT1, indicating a strong metabolic and cytoprotective effect that surpasses that of OMP.

### 3.12. Histopathological Evaluation

**Figure 10:**
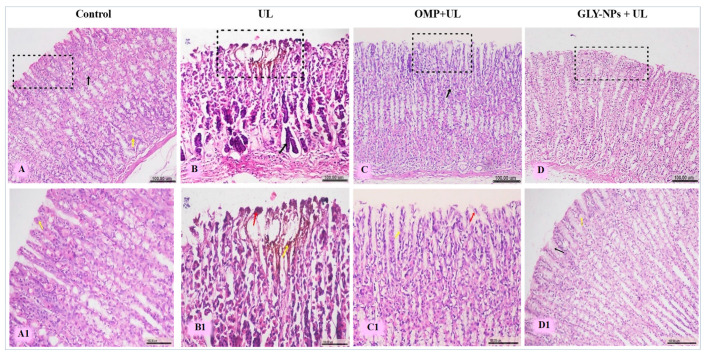
Histopathological examination of gastric mucosa and submucosa layers showing (**A**) control group normal fundic mucosa (black square), gastric pits (black arrow), and normal gastric gland (yellow arrow), (**A1**) normal epithelial layer (yellow arrow); (**B**) ulcerative lesions of epithelial layer (black square) and degenerated gastric gland (black arrow), (**B1**) hemorrhage (yellow arrow) and cell debris (red arrow); (**C**) desquamation on gastric mucosa (black square) and intact gastric pits (black arrow), (**C1**) erosion (yellow arrow) and inflammatory cells (red arrow); (**D**) preserved histological architecture of gastric tissue (black square), and (**D1**) layer of mucus on the surface (black arrow) and clear basal oval nuclei (yellow arrow). (H&E, scale bar: 100 µm).

The stomachs of the control group of rats had normal architectures, without any indications of mucosal damage (Figure 10A,A1). The UL group had histological changes in gastric tissue, including significant ulcerative lesions of the epithelial layer, defined by the loss of the tunica mucosa, exfoliated cells, and degeneration of gastric glands (Figure 10B). Furthermore, hemorrhagic gastritis and inflammatory cell infiltration were present (Figure 10B1). Conversely, the OMP + UL group exhibited slight enhancement of the fundic mucosa, characterized by inflammatory cell infiltration and desquamation of the stomach mucosa (Figure 10C,C1). Meanwhile, GLY-NPs + UL preserved the histological architecture of gastric tissue and exhibited a thick mucus layer on the surface, with surface cells abundant in mucus and displaying distinct basal oval nuclei (Figure 10D,D1).

Histological scoring indicated a significant elevation in gastric tissue damage in the UL group compared to the control (*p* = 0.001). The UL group had considerable mucosal damage, including epithelial disruption, inflammatory cell infiltration, and tissue necrosis. Treatment with OMP showed mild improvement with partial preservation of mucosal architecture and a non-significant reduction in histopathological scores compared to the UL group (*p* > 0.05). Similarly, the GLY-NPs + UL group exhibited moderate improvement, characterized by less inflammatory alterations and partial restoration of the epithelial lining; however, this enhancement was not statistically significant when compared to the UL group (*p* > 0.05). No substantial change was seen between the OMP- and GLY-NP-treated groups (*p* > 0.05). This result indicates a progressive healing process; however, complete restoration of the normal histological structure has not yet occurred (Supplementary Figure S4).

### 3.13. Immunohistochemical Evaluation of PTEN, PI3K, and AKT

**Figure 11:**
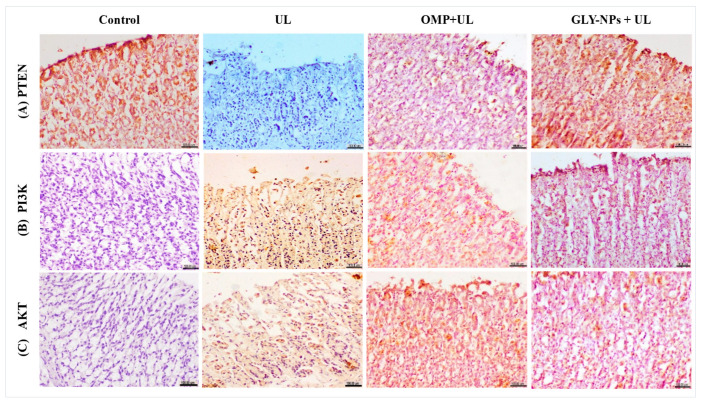
Immunohistochemical evaluation of (**A**) PTEN, showing (Control) high expression of PTEN, (UL) tunica mucosa cells of gastric showing absence of PTEN expression, (OMP + UL) weak cytoplasmic staining of gastric mucosa, and (GLY-NPs + UL) moderate expression of PTEN of lamina epithelialize; (**B**) PI3K, showing (Control) no expression of PI3K of tunica mucosa, (UL) tunica mucosa showing increased PI3K expression, (OMP + UL) abundant positive cells of gastric mucosa, and (GLY-NPs + UL) mild expression of PI3K of lamina epithelialize; (**C**) AKT, showing (Control) no expression of PI3K of tunica mucosa, (UL) tunica mucosa showing increased PI3K expression, (OMP + UL) abundant positive cells of gastric mucosa, and (GLY-NPs + UL) mild expression of PI3K of lamina epithelialize. (IHC, ×400. Scale bar: 100 μm).

Immunohistochemical evaluation of PTEN, PI3K, and AKT exhibited increased expression of PTEN, but PI3K and AKT showed no expression in the stomach mucosa of the control group. In contrast, the UL group demonstrated a notable increase in PI3K and AKT expression, accompanied by the absence of PTEN expression in the tunica mucosa cells. Conversely, the OMP + UL group had weak PTEN expression and moderate PI3K and AKT immunoreactivity in the gastric mucosa., while the GLY-NPs group exhibited substantial PTEN expression along the epithelial lining of the lamina propria, with only minimal expression of PI3K and AKT (Figure 11A–C). These findings suggest a more favorable modulatory effect of GLY-NPs on the PTEN/PI3K/AKT pathway compared to OMP.

Moreover, immunohistochemical scoring revealed significant downregulation of PTEN expression with a corresponding elevation of PI3K/AKT immunoreactivity in the UL group. The OMP + UL group showed a slight elevation in PTEN expression and a decrease in PI3K and AKT activity relative to the UL group; yet, these alterations did not achieve statistical significance when compared to the UL group (*p* > 0.05). Conversely, the GLY-NPs group exhibited a markedly elevated PTEN expression relative to the UL group (*p* < 0.05), alongside a more pronounced reduction in PI3K and AKT immunoreactivity; however, these alterations did not attain statistical significance when compared to the UL group (*p* > 0.05). The decrease in PI3K/AKT expression in the GLY-NPs group was more dramatic than that seen with OMP; nonetheless, the alterations were statistically non-significant, indicating an incomplete restoration of normal expression profiles (as shown in Supplementary Figure S5).

## 4. Discussion

The experimental model for ethanol-induced GUs elucidates the origins of these ulcers in humans, thereby helping the identification of the anti-ulcer properties of drugs and the potential molecular mechanisms involved in this process [45,46]. OMP, a widely used proton pump inhibitor, efficiently diminishes acid secretion but is frequently linked to adverse side effects, prompting the exploration of safer alternatives. GLY acts as an anti-inflammatory agent; nevertheless, the application of GLY is constrained by its inadequate bioavailability and low solubility [47]. These limitations can be mitigated by nanoparticle delivery methods, which have demonstrated efficacy in enhancing the solubility and stability of natural substances [48]. Therefore, the current study aimed to develop GLY-NPs to improve their therapeutic potential and to comparatively assess their efficacy against OMP in ethanol-induced GUs in Wistar rats.

A crucial determinant in evaluating nanoparticle performance is their size, which influences drug circulation and biodistribution [49]. The GLY-NPs exhibited a particle size of 136.3 nm. It is well established that nanoparticle size significantly influences their interaction with tissues and specific cellular structures, as well as their pharmacokinetics and clearance in nanoparticle-based drug delivery systems [50]. Moreover, GLY-NPs exhibited a PDI of 0.489. The PDI value indicates moderate variation in size distribution. Singh et al. [51] indicate that a PDI of less than 0.3 is typically preferred; however, values below 0.5 are also deemed acceptable. Similarly, Shimojo et al. [52] indicate that the ideal PDI is below 0.5, but PDI values above 0.5 indicate a considerably broad distribution.

Furthermore, zeta potential is a crucial metric for evaluating physical stability. Our results indicated that the GLY-NPs displayed a zeta potential of −19.2 mV, suggesting acceptable physical stability. In addition, the negative zeta potential, as reported in this study, signifies adequate electrostatic stabilization, crucial for averting agglomeration and improving stability [53]. Moreover, nanoparticles with an appropriate zeta potential prevent aggregation by producing repulsive forces and enhancing permeability across cell membranes [54]. The TEM studies confirmed that the GLY-NPs exhibited a spherical to oval shape, with sizes ranging between 50 and 135 nm. The reduction in size is essential for improving the solubility and bioavailability of GLY, since smaller particles augment the surface area, hence permitting increased interaction with the solvent and improving dissolution [55]. The XRD pattern of GLY-NPs displayed a less intense peak and diffuse peaks, indicating a loss of crystallinity and implying the development of an amorphous or semi-crystalline nanoscale formulation. Amorphous drugs generally have markedly higher apparent solubility than crystalline drugs due to their higher energy state, which promotes supersaturation in the gastrointestinal tract, hence improving bioavailability [56].

The in vitro release profile of GLY from the synthesized GLY-NPs exhibited sustained and controlled release characteristics over an 8 h duration. This release pattern is beneficial for sustaining therapeutic drug levels over an extended period [57], which may be especially effective for the treatment of GUs. Furthermore, the GLY-NPs demonstrated a high EE of 98.28 ± 0.23%, indicating potential for improving the therapeutic efficacy of GLY. EE% is a significant determinant influencing the efficacy and toxicity of nanoformulations [58]. Tong and Cheng [59] observed that insufficient EE% during nanoparticle production could limit drug loading and impair release control, thereby reducing therapeutic efficacy and elevating systemic adverse effects.

The administration of ethanol induces gastric mucosal injury and subsequent infiltration of inflammatory cells, diminishes gastric mucus formation, and lowers gastric juice pH [60]. Our findings demonstrated that the oral administration of ethanol led to significant hemorrhagic lesions on the gastric mucosa, a marked rise in stomach weight, stomach coefficient, and UI%, while concurrently reducing body weight and gastric pH values. Numerous investigations have documented similar effects of ethanol consumption on the gastric mucosa [61,62,63]. Conversely, the administration of OMP or GLY-NPs significantly preserved gastric mucosa, exhibiting minimal ulcerative lesions and reduced edema, accompanied by a notable decrease in stomach weight, stomach coefficient, and UI%, while enhancing body weight and ulcer inhibition (%) in comparison to the UL group. Moreover, GLY-NPs demonstrate a more pronounced action in this context. Additionally, OMP administration markedly increased gastric pH relative to the ulcer group, but GLY-NPs seemed to mildly enhance the pH in comparison to the ulcer group. The data indicate that although GLY-NPs produce a slight alkalinizing impact, their principal mechanism of action is mucosal protection and anti-inflammatory benefits rather than direct acid suppression.

The liver serves as the primary organ for toxic substances, whereas the kidney operates as a metabolic organ responsible for waste elimination and fluid balance. In our study, ethanol-induced GUs in rats caused a marked deterioration of renal function, indicated by considerable increases in serum creatinine, urea, and uric acid concentrations. Furthermore, it was linked to a notable decline in liver function, as indicated by increased serum levels of ALT, AST, and ALP. Consistent with our findings, Ghareeb et al. [61] revealed that the ethanolic groups had liver and kidney dysfunction, evidenced by markedly raised serum hepatorenal biomarkers, including AST, ALT, urea, and creatinine. Conversely, our findings indicate that serum levels of ALT, AST, ALK, urea, uric acid, and creatinine in the OMP + UL or GLY-NPs + UL groups were considerably reduced compared to the UL group, suggesting a potential protective impact. Moreover, the findings confirm that GLY-NPs demonstrate superior hepatoprotective effects and renal function preservation compared to OMP in the context of ethanol-induced GUs. These findings align with prior studies demonstrating the nephroprotective effects of GLY in various nephrotoxic models, including cisplatin-induced renal damage [64], indicating that GLY formulations may provide multi-organ protective advantages.

Our findings revealed considerable hormonal disturbances, evidenced by a marked increase in serum gastrin and somatotropin levels in the UL group. Consistent with our findings, Sun et al. [65] reported elevated gastrin levels in the plasma of rats with GUs. These elevations signify pathological alterations linked to mucosal injury, elevated gastric acid secretion, and a systemic stress reaction. The administration of OMP or GLY-NPs markedly diminished gastrin and somatotropin levels compared to the UL group, with a considerably higher reduction observed with GLY-NPs than with OMP. This enhanced effect signifies improved bioavailability and targeted distribution using nanoparticles, enhancing hormonal balances in GUs.

Oxidative stress and inflammation are recognized as the primary contributors to ethanol-induced GUs [66]. The gastric damage induced by ethanol is significantly linked to elevated production of ROS, which promotes lipid peroxidation and oxidative stress in gastric tissues [46]. This process results in the excessive generation of MDA, a principal biomarker for evaluating oxidative stress in alcohol-related injury models of gastric tissues [67]. Furthermore, excessive accumulation of ROS can exhaust endogenous antioxidants and impair internal defense mechanisms [68]. The findings align with our results, which revealed markedly elevated levels of MDA in ethanol-induced gastric tissues in the UL group, signifying increased lipid peroxidation, whereas the levels of CAT, SOD, and GSH-Px were substantially diminished in ethanol-induced GUs, indicating compromised antioxidant defense. Furthermore, comparable detrimental effects of ethanol on stomach tissues have been noted in many rat investigations [69,70].

Conversely, the administration of OMP or GLY-NPs markedly decreased MDA levels while dramatically elevating CAT, SOD, and GSH-PX levels, with a more pronounced effect observed with nano administration. The results indicate that GLY-NPs confer gastroprotective benefits via diminishing oxidative stress and stimulating the formation of antioxidant enzymes. The antioxidative impact of the nanomaterial demonstrated in this work may be ascribed to improved bioavailability and targeted delivery. Zhao et al. [26] reported similar antioxidative properties, revealing that GLY nanoparticles displayed antioxidant effects in both in vitro and in vivo studies. The observed effects were associated with the substantial antioxidant activity of GLY, which is attributed to several isoflavonoids [71].

Besides oxidative stress, inflammation is crucial in the development of ethanol-induced GUs. Experimentally, ethanol administration destroys the mucosal layer, damages cellular surfaces, and disrupts intercellular connections, leading to inflammatory responses, mucosal bleeding, submucosal edema, and gastric mucosal ulcers [72]. Damage to the gastric mucosa triggers an inflammatory response, attracting immune cells that release inflammatory mediators like TNF-α, which subsequently obstructs microcirculation at the ulcer margins, promotes cellular proliferation, and impairs vascular regeneration, thereby delaying ulcer healing [73,74,75]. This cascade initiates the MAPK pathway, resulting in the programmed cell death of gastric epithelial cells [76]. Additionally, p38 MAPK functions as an upstream signal in the inflammatory pathway, promoting the activation of the NF-κB signaling pathway [77]. In contrast, IL-10 demonstrates anti-inflammatory properties by diminishing the expression of inflammatory mediators such as TNF-α. These findings align with our results, which indicated that ethanol-induced ulcers exhibited raised levels of inflammatory markers such as NF-κB, TNF-α, and p38 MAPK, while IL-10 levels were considerably reduced in gastric tissue affected by ethanol.

However, our data indicated that the administration of either OMP or GLY-NPs resulted in a significant decrease in NF-κB, TNF-α, and p38 MAPK levels, whereas IL-10 levels were dramatically elevated. Significantly, GLY-NPs demonstrated more substantial effects in this context than the reference drug. Zhao et al. [26] revealed that GLYNPs significantly inhibited inflammatory cytokines in their research, which examined GLYNPs as potential therapeutic agents for COVID-19. Additionally, a similar study conducted by Zeeshan et al. [39] regarding GLY-encapsulated pH-sensitive poly-(lactic-co-glycolic acid) nanoparticles demonstrated a substantial decrease in inflammation and facilitated the healing of bowel mucosa in an inflammatory bowel disease model, thereby reinforcing the therapeutic potential of GLY-NPs in inflammatory disorders. Furthermore, prior studies indicate that increased antioxidant levels inhibit NF-κB activation induced by ROS and obstruct the synthesis of several cytokines [78], a finding that is supported by our results, which demonstrated a similar inhibitory effect on NF-κB signaling. Moreover, Wang et al. [79] demonstrated that GLY nanoparticles more effectively inhibited the production of lipopolysaccharide-induced inflammatory cytokines, such as TNF-α, in macrophage cells compared to unprocessed GLY, highlighting the superior anti-inflammatory efficacy of the nanoparticle formulation.

We further demonstrated an important role of the TGF-β1/Smad3 and JAK/STAT3 pathways in the pathogenesis of GUs in our study. The TGF-β/Smad pathway is associated with the progression of fibrosis in several animal models [80,81,82]. The stimulation of TGF-β1 induces fibrosis via augmenting TGF-β/Smad signaling pathways, leading to the activation of myofibroblasts and the synthesis of extracellular matrix components [83]. Upon activation of the signaling pathway, Smad proteins elicit a spectrum of biological responses, encompassing both pro-fibrotic and anti-fibrotic effects. Moreover, intricate connections exist between TGF-β/Smad and other signaling pathways [84]. Intercommunication between the TGF-β/SMAD and JAK/STAT pathways has also been reported [85,86,87]. The JAK/STAT pathway is crucial in modulating inflammatory responses, and its dysfunction can promote the development of GUs. This intracellular transduction pathway is crucial in numerous biological processes, with its activation associated with a range of immunological and inflammatory diseases [88]. Although its dysregulation has predominantly been examined in ulcerative colitis [89,90], this underscores the significance of JAK/STAT regulation in controlling inflammatory diseases.

Our data suggest a potential involvement of these pathways in GU pathogenesis, as evidenced by a significant increase of both TGF-b/SMAD and *JAK2*/*STAT3* in the UL group relative to the control (*p* < 0.0001). Conversely, administration of OMP or GLY-NPs exhibited an anti-fibrotic impact, proven by a considerable reduction in Smad3 and TGF-β1 levels, with a more prominent effect in the GLY-NPs-treated group. Moreover, both treatments suppressed the activation of *JAK2*/*STAT3*, with GLY-NPs demonstrating a more pronounced reduction in pathway activation, suggesting a potentially greater modulatory effect of GLY-NPs on inflammation-related signaling. The augmented effect may result from the improved bioavailability of GLY-NPs, providing more effective regulation of inflammatory pathways at the cellular level. Consequently, the targeted suppression of the TGF-β1/Smad3 and JAK2/STAT3 signaling pathways may serve as a viable therapeutic objective for the mitigation of GUs.

On the other hand, the SIRT-1/PGC-1α/FOXO1 pathways are essential in protecting stomach tissue against oxidative stress and inflammation, which are significant contributors to GU formation. SIRT1 modulates the inflammatory response via influencing FOXO1/NF-κB [91,92,93]. Additionally, SIRT-1 inhibits NF-κB activation, which plays a critical role in regulating oxidative stress and inflammation [9], and may diminish the cellular ROS load [94]. Moreover, SIRT1 may directly affect the functionality of PGC-1α [95], potent enhancers of mitochondrial respiration and gene transcription that can diminish oxidative stress and inflammation [96]. Similarly, this study demonstrated that the levels of SIRT-1, PGC-1α, and FOXO1 were decreased in response to ethanol-induced ulcers. The results align with earlier findings by Alamoudi et al. [97], who evidenced ethanol-induced downregulation of the SIRT-1/PGC-1α pathway in stomach tissue. These results further corroborate previous findings [96,98].

Conversely, administration of OMP or GLY-NPs resulted in the activation of SIRT-1, PGC-1α, and FOXO1, with GLY-NPs exhibiting a more significant impact. The data demonstrate that GLY-NPs substantially mitigate the ethanol-induced downregulation of FOXO1, PGC-1, and SIRT1, indicating a pronounced metabolic and cytoprotective effect that exceeds that of OMP. Nanoparticle compositions frequently enable regulated and sustained drug release, preserving therapeutic levels for extended durations compared to conventional formulations. Furthermore, improved bioavailability results in superior absorption and delivery to the targeted stomach regions in comparison to conventional medications such as OMP.

The phosphatase and tensin homolog (PTEN)/phosphatidylinositol3-kinase (PI3K)/protein kinase B (AKT) pathway is crucial for regulating several cellular activities. PTEN is intricately associated with the activation of the PI3K/AKT signaling pathway in human stomach tissue [99]. The loss of PTEN function results in the activation of the PI3K/Akt pathway, subsequently phosphorylating downstream signaling proteins to promote cell growth and proliferation [100]. Activated Akt phosphorylates substrates with serine/threonine residues, exerting many biological effects, including enabling the release of inflammatory cytokines and inhibiting cell death [101,102]. In a comparable manner, our results from immunohistochemical staining revealed that the ulcer-induced group demonstrated a marked downregulation of PTEN expression, alongside an increase of PI3K/AKT immunoreactivity, signifying the activation of the PI3K/AKT pathway. The administration of OMP resulted in a modest enhancement of PTEN expression and a decrease in PI3K/AKT activity. The delivery of GLY-NPs led to a more pronounced trend toward normalization in marker expression. The influence of GLY-NP on PTEN expression may be ascribed to its capacity to downregulate oxidative stress and inflammatory mediators, which likely promote the restoration of PTEN expression and the subsequent modification of the PI3K/Akt pathway.

### Study Strengths and Limitations

This study comprises strengths, including the development of GLY-NPs to enhance therapeutic efficacy. The formulation was subjected to thorough investigation employing diverse methodologies. The study incorporated biochemical, molecular, and histological assessments to analyze the therapeutic effects, yielding a comprehensive understanding of their mode of action. Nonetheless, the study possesses limitations. The in vitro release study was inadequately confirmed and lacks kinetic analysis, limiting comprehension of the release mechanism and its in vivo relevance. A significant limitation is the restricted number of animals per group. Another limitation is the short duration of treatment (7 days pre-treatment plus 7 days post-ethanol administration), which is intended to evaluate the protective and therapeutic effects of GLY-NPs. Nonetheless, this duration may be inadequate for a comprehensive evaluation of mucosal regeneration or the sustained anti-ulcer effectiveness. No prolonged follow-up was performed, hence constraining conclusions regarding enduring healing or chronic effects. Future research with extended treatment and follow-up durations is necessary to examine these factors.

## 5. Conclusions

The current study demonstrates that the GLY-NPs created exhibited anti-gastric ulcer effects in ethanol-induced GUs in Wistar rats. The anti-gastric ulcer effects were attributed to the reduction of oxidative stress and inflammatory markers. Moreover, GLY-NPs modulate the JAK2/STAT3 and TGF-β1/Smad3 signaling pathways while enhancing the protective pathway SIRT1/FOXO1/PGC-1α, resulting in improved histology outcomes compared to conventional OMP therapy. The data indicate that GLY-NPs may be a viable alternative treatment for gastric ulcers; nevertheless, further study is required to confirm their therapeutic efficacy.

## Figures and Tables

**Table 1:** Primer sequence for all studied genes.

	Forward Sequence	Reverse Sequence	Genes Accession Numbers
*STAT3*	AGAGGCGGCAGCAGATAGC	TTGTTGGCGGGTCTGAAGTT	NM_012747.2
*JAK2*	CAATGATAAACAAGGGCAAATGAT	CTTGGCAATCTTCCGTTGCT	NM_031514.1
*GAPDH*	TGGATTTGGACGCATTGGTC	TTTGCACTGGTACGTGTTGAT	NM_017008.4

*STAT3*, signal transducer and activator of transcription 3; *JAK2*, Janus kinase 2; and *GAPDH*, glyceraldehyde-3-phosphate dehydrogenase.

**Table 2:** Serum gastrin and somatotropin levels in ethanol-induced GUs in rats.

	Control	UL	OMP + UL	GLY-NPs + UL
Gastrin (pg/mL)	99.69 ± 2.68	420.5 ± 5.92 ^a^	254.0 ±2.86 ^ab^	170.8 ± 5.57 ^abc^
Somatotropin (pg/mL)	26.63 ± 2.75	215.3 ± 5.23 ^a^	151.6 ± 2.67 ^ab^	88.62 ± 2.07 ^abc^

Data are presented as mean ± SEM (n = 6). One-way ANOVA, followed by Tukey’s post hoc test, was performed to assess statistical significance across all groups. *p* < 0.05 was considered statistically significant. Superscripts: ^a^: significant vs. control group (*p* < 0.05), ^b^: significant vs. UL group (*p* < 0.05), and ^c^: significant vs. to the OMP + UL group.

## Data Availability

Data will be made available upon request.

## Supplementary Materials

The following supporting information can be downloaded at https://www.mdpi.com/article/10.3390/antiox14080990/s1.

## Abbreviations

The following abbreviations are used in this manuscript:

GUs	Gastric Ulcers
OMP	Omeprazole
GLY	Glycyrrhizic Acid
GLY-NPs	Glycyrrhizic Acid Nanoparticles
ROS	Reactive Oxygen Species
MDA	Malondialdehyde
TNF-α	Tumor Necrosis Factor-Alpha
SOD	Superoxide Dismutase
MAPKs	Mitogen-Activated Protein Kinases
NF-κB	Nuclear Factor Kappa B
JAK	Janus Kinase
STAT	Signal Transducer and Activator of Transcription
IL-10	Interleukin-10
TGF-β	Transforming Growth Factor-Beta
PTEN	Phosphatase and Tensin Homolog
PI3K	Phosphatidylinositol 3-Kinase
AKT	Protein Kinase B
SIRT1	Sirtuin 1
PGC-1α	Peroxisome Proliferator-Activated Receptor Gamma Coactivator Alpha
GSH-Px	Glutathione Peroxidase
