# Supplementary material for: Development of Glycyrrhizic Acid Nanoparticles for Modulating Gastric Ulcer Healing: A Comparative In Vivo Study Targeting Oxidative Stress and Inflammatory Pathways
Source: Antioxidants (Basel). 2025 Aug 12;14(8):990. doi: 10.3390/antiox14080990 (PMC12382799; doi:10.3390/antiox14080990)
Supplement: Supplementary file 1 [file antioxidants-14-00990-s001.zip › antioxidants-3767044-supplementary.pdf]

Supplementary Data

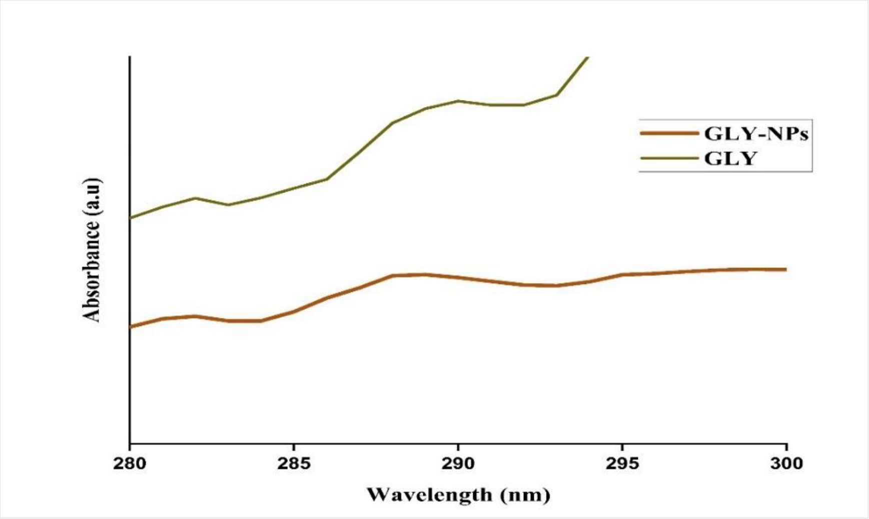

Supplementary Figure S1: UV Spectra of GLY and GLY-NPs.

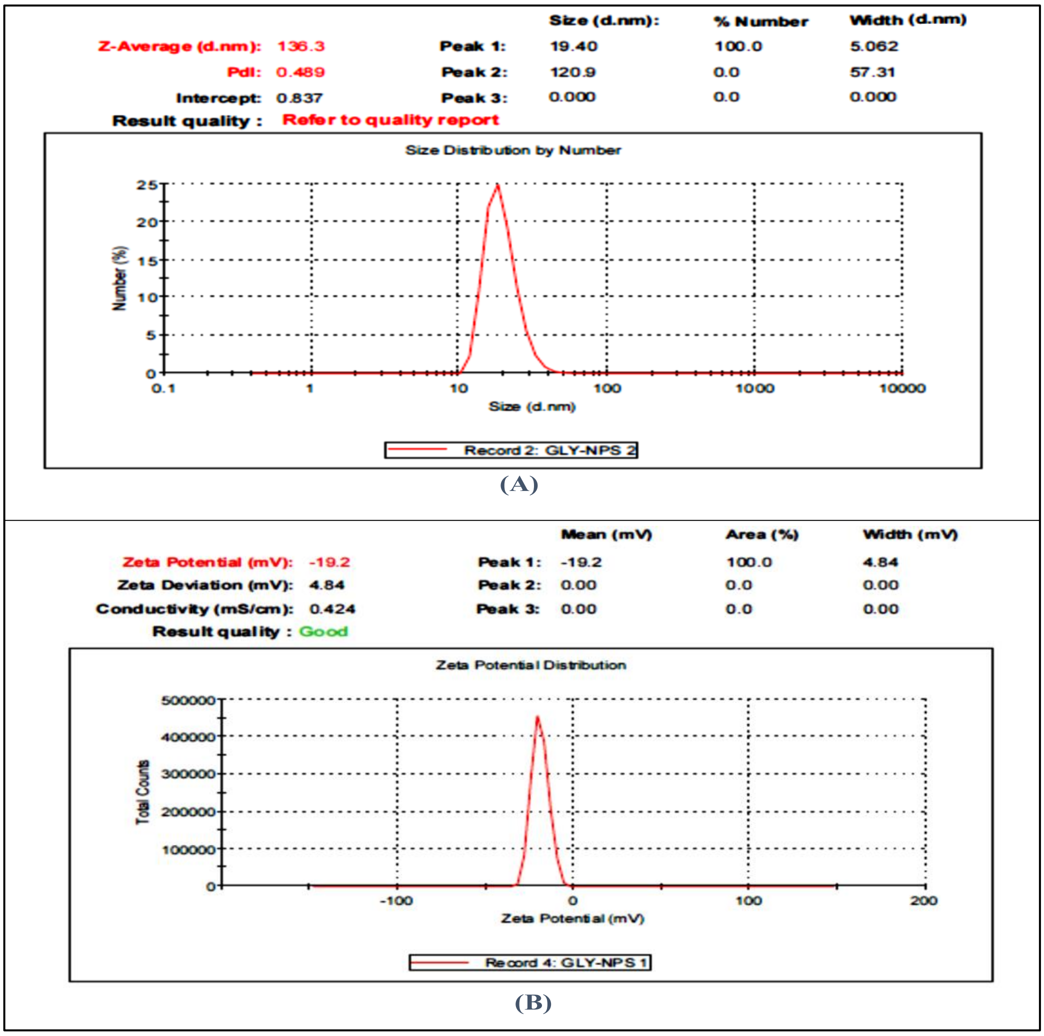

Supplementary Figure S2. (A) Particle size and PDI of the GLY-NPs. (B) Zeta potential analysis of the GLY-NPs.

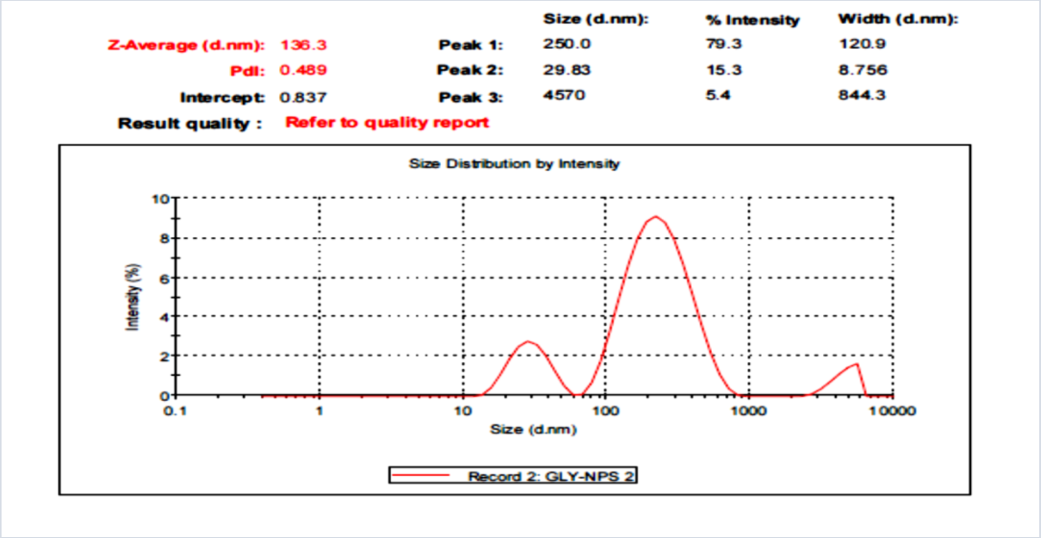

Supplementary Figure S3. Size Distribution of the GLY-NPs Report by Intensity.

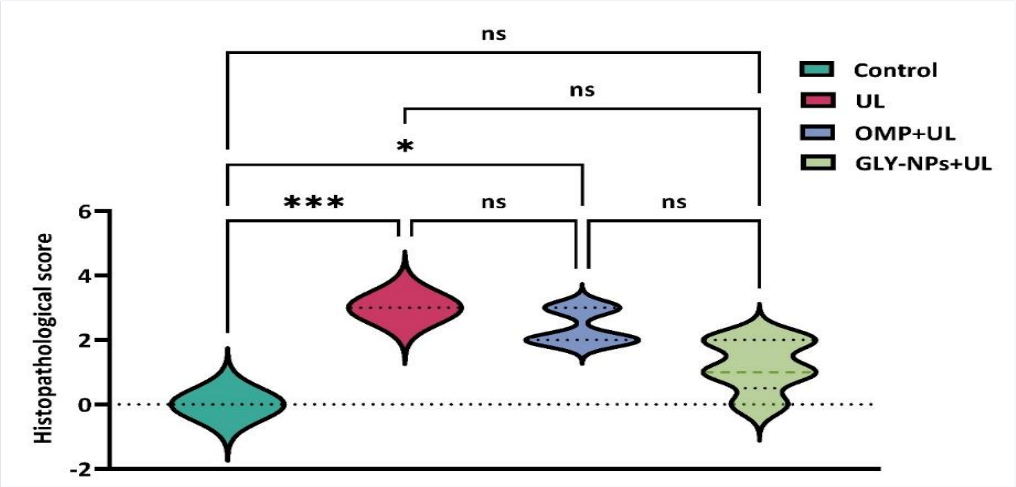

Supplementary Figure S4: Representative Histopathological Scores of Gastric Tissue in Different Experimental Groups. Data are expressed as median with interquartile range (IQR) (n=6). Statistical significance was assessed by Kruskal-Wallis test followed by Dunn's multiple comparisons test. Significance levels are indicated as: ***p < 0.001, *p < 0.05, ns = not significant.

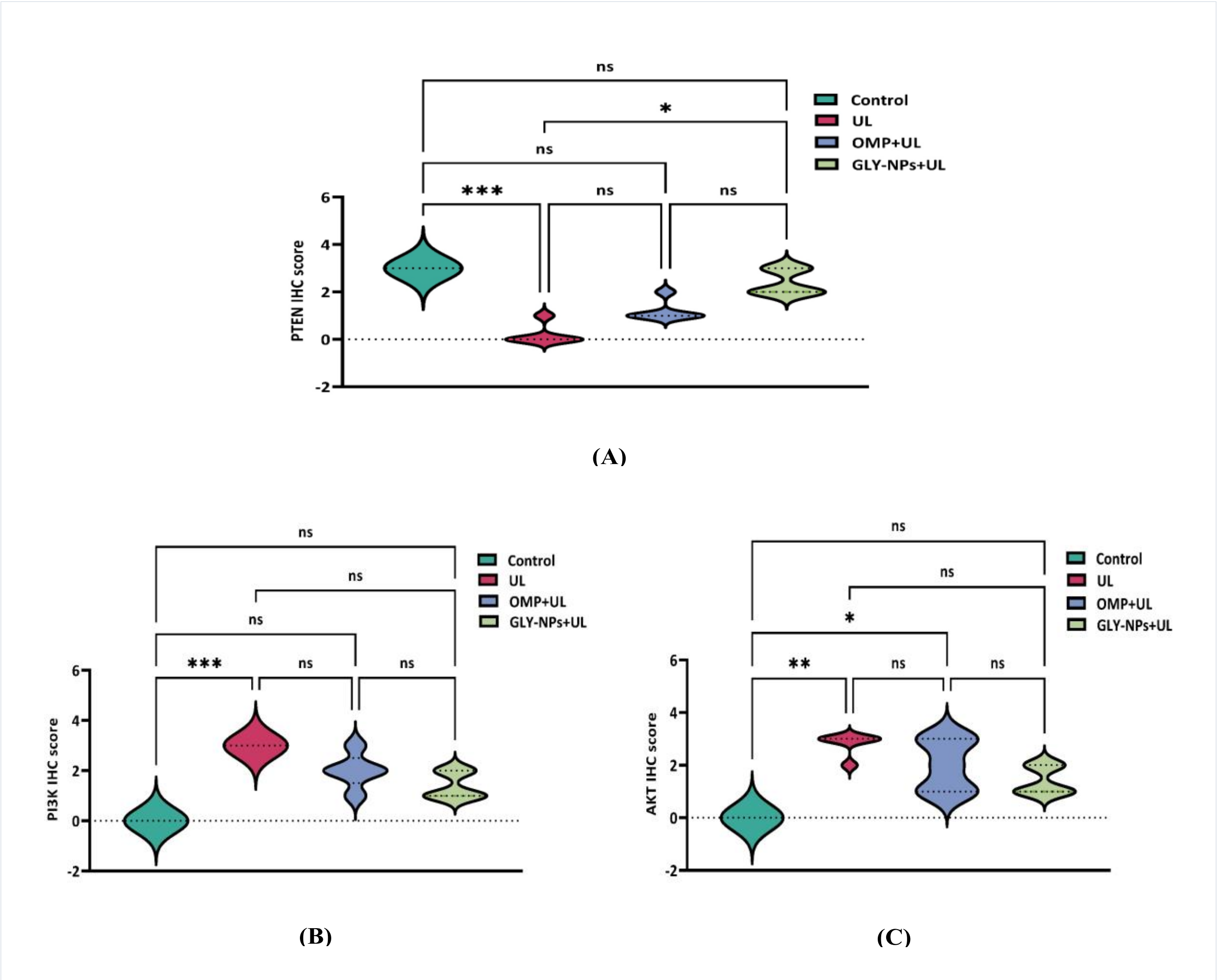

Supplementary Figure S5: Immunohistochemical Scoring of PTEN, PI3K, and AKT in Gastric Tissues. Data are presented as mean ± SEM (n = 5). Statistical significance was assessed by the Krus-kal-Wallis test followed by Dunn's multiple comparisons test. p < 0.05 was considered statistically significant. ***p < 0.001, **p < 0.01, *p < 0.05, ns = not significant
